# ‘So Let's Go On Like This?’—Shared Decision‐Making and the Use of Outcome Information in Routine Care Management for People With Multiple Sclerosis

**DOI:** 10.1111/hex.70009

**Published:** 2024-10-24

**Authors:** Olga C. Damman, Laxsini Murugesu, Vincent de Groot, Brigit A. de Jong

**Affiliations:** ^1^ Department of Public and Occupational Health and Amsterdam Public Health Research Institute, Quality of Care Amsterdam UMC location Vrije Universiteit Amsterdam Amsterdam The Netherlands; ^2^ Department of Rehabilitation Medicine MS Center Amsterdam, Amsterdam Neuroscience and Amsterdam Movement Sciences Research Institute, Rehabilitation & Development, Amsterdam UMC location Vrije Universiteit Amsterdam Amsterdam The Netherlands; ^3^ Department of Neurology MS Center Amsterdam, Amsterdam Neuroscience and Amsterdam Public Health Research Institute, Quality of Care, Amsterdam UMC location Vrije Universiteit Amsterdam Amsterdam The Netherlands

**Keywords:** multiple sclerosis, outcome information, patient‐reported outcome measures, shared decision‐making

## Abstract

**Introduction:**

This study aimed to investigate how shared decision‐making (SDM) and the use of different types of outcome information are applied in routine care management for people with multiple sclerosis (MS) in an academic outpatient clinic.

**Methods:**

This qualitative study used the following: (a) observations of clinical encounters (*N* = 23) between patients and healthcare professionals (HCPs), (b) interviews with those patients (*N* = 17) and (c) interviews with those HCPs (*N* = 7). HCPs were not trained in SDM before the study. Audio recordings were transcribed literally. Transcriptions were analysed using qualitative thematic analysis.

**Results:**

Outcome information was hardly discussed with patients, apart from clinical outcome information at an individual level, such as MRI or lab results. This use of clinical outcome information did not automatically lead to a process of SDM. HCPs tended to implicitly present choices to patients after signalling and discussing ‘problems’. In the interviews, patients indicated that they tended to consent to the advice given by HCPs and to prefer not too much change in treatment plans. However, they also emphasized the importance of being informed about available options with benefits and harms. We observed multiple discussions about patients' preferences, especially related to patients' experiences and priorities.

**Conclusions:**

Overall, SDM and the use of different types of outcome information did not seem to be enacted in routine care management for people with MS, mostly because choices were not explicitly mentioned or discussed. However, discussions about patients' experiences and priorities did take place. Training HCPs further and developing patient information seem reasonable steps to proceed.

**Patient or Public Contribution:**

People with MS contributed as research participants and provided us with their experiences in interviews. Furthermore, representatives of two patient organizations contributed to the study by reviewing the interview protocol for people with MS.

## 1 Introduction

In the management of chronic diseases such as multiple sclerosis (MS), shared decision‐making (SDM) has been increasingly advocated to achieve patient‐centred and outcome‐driven care [1, 2]. SDM is traditionally defined as a collaborative way of decision‐making in which healthcare professionals (HCPs) and patients make healthcare decisions together, based on the best scientific evidence available, the HCP's experience and the patient's values and preferences [3, 4]. Such collaborative decision‐making is especially important when treatment options with uncertain effects have to be weighed. SDM has also been described in relation to signalling and managing different problematic situations [5], such as those that may appear in chronic disease management after first‐time decisions about treatment have been made. Apart from RCT‐derived evidence, healthcare systems are also gradually implementing the use of other types of numerical evidence or so‐called ‘outcome information’ to facilitate SDM, such as information from patient‐reported outcome measures (PROMs). PROMs are standardized questionnaires measuring the patients' views of their health status [6], including their symptoms, physical, mental and social functioning and overall quality of life. Using PROMs at both the individual (*N* = 1) and aggregated (group) levels, the idea is that its use will increase early detection of symptoms, discussion of patients' needs to manage those symptoms and subsequent SDM and information provision about diagnostic and treatment options [7, 8, 9].

There is some evidence, mainly from oncology, that the application of PROMs in routine clinical care is associated with positive outcomes, such as increased discussion of psychosocial issues, improved communication between HCPs and patients, higher patient satisfaction and even increased quality of life and clinical outcomes [10, 11, 12]. However, there are also known barriers to implementing PROMs [13, 14]. For example, problems related to information technology have been widely documented, as well as the perceived burdens of completing and using PROMs by both HCPs and patients [13, 15, 16, 17]. Therefore, the question remains whether these other types of numerical outcome information are actually used for decision‐making in the clinical encounter. In a recent study by Westerink et al. [17], HCPs reported finding it challenging to translate outcome data into treatment decision‐making. Importantly, research in this field has mainly concentrated on one‐time decisions such as in oncology, which are arguably different from decisions made in the context of chronic progressive diseases. For example, for patients with MS, there is usually a first‐time ‘big’ decision about disease‐modifying treatment (DMT), with a series of consecutive ‘smaller’ decisions depending on symptoms and treatment side effects experienced [18]. Especially for the consecutive decisions made in routine care management, the use of PROMs information may be valuable. Furthermore, patients are relatively young and usually experience fear and uncertainty about transitioning to progressive forms of their disease [18, 19, 20]. This may result in a process in which the treatment plan is reconsidered and treatment options are again weighed using aggregated PROMs data available.

SDM as practiced in a clinical encounter is thought to consist of different key steps or elements, although they do not necessarily occur in chronological order. The four‐step SDM model applied in the current study as a framework (see Box 1) consists of the following: (1) choice, (2) options, (3) preferences and (4) decision [21]. Before the widespread implementation of PROMs through dashboarding and other technology, talking with patients about options in SDM was mainly informed by RCT‐derived evidence at the group level, while discussing patients' preferences by the elicitation of patients' values and priorities. Both aspects (options and preferences) can be addressed in the clinical encounter, but also in Patient Decision Aids (PtDAs) [22, 23, 24, 25]. Despite PROMs being collected for routine care in MS care [23] and scholars arguing for the use of PROMs to make SDM more individualized [8, 26, 27], how SDM and the use of outcome information are enacted in the setting of routine care management for people with MS is still unknown.

BOX 1 **The four‐step SDM model used in the current study.**

Synthesizing different definitions and models of shared decision‐making (SDM) in the research literature, Stiggelbout, Pieterse, and De Haes [21] distinguished four steps in an SDM process. The four steps are roughly the same as the steps distinguished earlier by Elwyn and colleagues, except that the third step in Elwyn's model was separated into two distinct steps by Stiggelbout and colleagues. The four steps are as follows:

1. Choice: The professional informs the patient that a decision is to be made and that the patient's opinion is important.
2. Options: The professional explains the options and their pros and cons.
3. Preferences: The professional and the patient discuss the patient's preferences and the professional supports the patient in deliberation.
4. Decision: The professional and patient discuss the patient's wish to make the decision, make or defer the decision and discuss follow‐up.

Note that the labels Choice, Options, Preferences and Decision were applied by the authors of this paper and not by Stiggelbout and colleagues in their original paper. This application of labels to the different steps has been done previously in the Netherlands and is often referred to as the four‐step SDM model (e.g., see 4-stappenmodelSamenbeslissen|Opleidingsmateriaal|uitkomstgerichtezorg).

### 1.1 Study Aim and Research Questions

This study aimed to investigate how SDM and the use of diverse types of ‘outcome information’ including PROMs are applied in routine care management for people with MS, with the setting of our academic centre as a case example. In doing so, we did not focus on the ‘big’ decision about the use of DMT, but explicitly on dealing with potential issues and problems in chronic care management afterwards, and the often ‘smaller’ consecutive decisions that may follow. Our primary research question (RQ1) was: ‘How do HCPs use diverse types of outcome information on an individual (*N* = 1) level and aggregated (group) level in clinical encounters with patients, and which SDM steps/elements are thereby supported?’ Previous research in both SDM [28, 29, 30] and PROMs [31, 32] indicates that patients from underserved populations or lower health literacy seem to be falling behind. Therefore, the secondary research question (RQ2) was: ‘How do these processes potentially differ for lower health literate patients versus higher health literate patients?’

## 2 Methods

### 2.1 Study Design

This study had a qualitative research design and consisted of the following: (a) observations of clinical encounters (*N* = 23) between people with MS (referred to as ‘patients’) and their HCP, (b) interviews with those patients (*N* = 17) and (c) interviews with those HCPs (*N* = 7). The observational data collected constituted the primary source of data, whereas interview data were additionally used to gain the perspectives of patients and HCPs. The observational study consisted of audio recordings of consultations between HCPs and patients. Audio recordings were chosen above video recordings because video recordings might hamper the participation of patients. Patients were invited to participate in subsequent interviews to explore their experiences with the consultation. All data were collected within one specific outpatient clinic, which was the Department of Neurology and Rehabilitation of the MS Center Amsterdam of Amsterdam UMC*.* As such, our study can be read as an academic centre's early experience with the use of diverse types of outcome information in SDM. The specific PROMs used at the outpatient clinic were the PROMIS Computerized Adaptive Tests (CATs) [33, 34] for fatigue, depression, anxiety, physical functioning, pain, sleep and ability to participate in social roles and activities.

### 2.2 Study Population and Recruitment

Patients were recruited directly by neurologists and rehabilitation physicians working in the MS centre. Inclusion criteria were as follows: (a) MS diagnosis following the McDonald 2017 criteria [35], (b) being 18 years or older and (c) previously signed informed consent to be willing to participate in the Amsterdam MS cohort study of our academic institution Amsterdam UMC. An exclusion criterion was having other neurological diseases or severe psychiatric disorders. Purposive sampling was conducted to include patients with varying health literacy levels; this was done by instructing clinicians to signal potential low health literacy‐related problems such as lower socioeconomic circumstances or ad hoc problems with understanding the information provided. We further instructed clinicians to recruit at appointments where regular care management took place, often following regular check‐ups. The researchers assisted in recruitment by signalling consultations with patients who had filled in PROMs. Eligible patients were informed about the study by telephone and sent an informational letter in plain language (in Dutch: ‘B1’). Informed consent was obtained by e‐mail and/or post approximately 2 weeks before the consultation. A non‐response rate could not be calculated because we did not want to burden the HCPs with extra administration.

### 2.3 Procedure/Data Collection

#### 2.3.1 Patients

After receiving informed consent from the patient, the HCP was asked to record the next consultation with this patient. Patients were thanked for participation and received a gift voucher of €10. The consultations were recorded in the period between 10 May 2022 and 19 October 2022.

Thereafter, patients were invited for an interview by the researchers by phone or video call. Patients invited first completed a health literacy assessment, to ensure inclusion of patients with lower health literacy. An interview protocol was used to guide the interviews (File S1). The interview started with an open‐ended question about how the patient had been doing since the recorded consultation. The interviewer then briefly summarized that consultation and asked if the patient agreed with this summary. Next, open‐ended questions were posed about general experiences with the consultation and about the outcome information communicated, for example, what they remembered best from the consultation (recall), and what (other) types of information the HCP had discussed with the patient. In this phase, we used standard probing questions to facilitate patients to express more experiences. After that, the interviewer provided additional examples of information (e.g., MRI result, treatment plan and PROMs scores) that had been provided in the consultation, in case the patient did not mention it him/herself. We asked patients what they remembered from this and if they could explain the main message of this information in their words. The next part of the interview consisted of questions about the patient's experiences and preferences in SDM in general as well as in the specific consultation. The interview ended with a question about what information the patient considered most useful in SDM about options. After the interview, patients received a questionnaire to assess sociodemographic background characteristics. File S2 provides the questions from this questionnaire as well as the health literacy assessment that was done before inclusion. All interviews were conducted by one researcher (L.M.) who was a female PhD student in health literacy‐sensitive SDM at the time of the study. She had received training in qualitative research methods during her PhD project.

Health literacy was assessed using the Newest Vital Sign in Dutch (NVS‐D) [36] and/or the Set of Brief Screening Questions (SBSQ) [37]. The exact assessment depended on how patients were interviewed (telephone or video call) and if they had time to complete it before or after the interview. As a result, some patients only completed the NVS‐D, some completed only the SBSQ and some completed both. Patients were thanked for participation and received another gift voucher of €15 for the interview. The duration of the interviews varied from 25 to 56 min. All interviews were conducted between 9 June 2022 and 28 October 2022.

#### 2.3.2 HCPs

After completing the data collection among patients, HCPs involved in the recorded consultations were invited to participate in an interview. We also used an interview protocol to guide these interviews (File S1). The interview started with a brief introduction of SDM. HCPs were asked about their attitude towards and their experiences with SDM and about potential decisions within the context of chronic care management potentially suitable for SDM. Next, they were asked what types of (numerical) evidence they usually discuss with patients in their consultations, and if not mentioned, the use of PROMs specifically. After these open‐ended questions, we inferred the different steps of SDM one by one and asked HCPs what types of outcome information they (might) use to facilitate those steps. The interviewer also asked about experiences with patients with lower health literacy. Subsequently, we introduced the recorded consultations and asked HCPs about their experiences with those consultations, in general and in relation to SDM. For each consultation, the interviewer provided the HCP with examples of decisions discussed and information provided, and the HCP was asked about his/her experiences and rationale for providing information in a certain way. HCPs were thanked for participation. The duration of the interviews varied from 36 to 52 min. All interviews were conducted between 7 October 2022 and 15 November 2022.

### 2.4 Analyses

Observations and interviews were transcribed verbatim and thematically analysed [38] using a mixture of inductive and deductive coding in the software programme MAXQDA. No transcriptions were returned to participants; for the patients, this was mainly to not burden them. Based on the audio recordings of the consultations, two researchers (L.M. and O.D.)—both experienced in SDM research—first independently performed open coding of data from two consultations and categorized those codes into a coding tree, according to the four‐step SDM model that included an additional first step about the use of PROMs [8]. Other parts of the consultation not directly related to the four‐step SDM model were also coded. Specific attention was paid to potential health literacy‐related issues [39]. Next, this process was repeated for the interviews with patients and HCPs involved in the two consultations. After reaching consensus about the coding structure, one researcher (L.M.) coded all other transcripts. She also used ‘memo's’ to make summaries of the consultations and interviews from the researchers' perspectives [38]. Based on the assigned codes, the two researchers (L.M. and O.D.) both independently identified underlying themes and subthemes in several meetings, and they also discussed data saturation. These themes were discussed within the research team until a consensus was reached.

## 3 Results

A total of 23 patients were included, and their consultations were audio‐recorded. Participants' characteristics are described in Table 1. Box 2 lists the ‘decision situations’ that we observed in the consultations.

**Table 1:** Participants' characteristics.

	*n* (%)	Mean (SD; range)
Age		67.7 (11.0; 21−70 years)
Gender		
Female	18 (78)	
Male	5 (22)	
Type of MS	—	
CIS		
RRMS		
SPMS		
PPMS		
Don't know		
Years since diagnosis		13.3 (7.8; 0−27 years)
Educational level	3 (13)	
Low	6 (26)	
Medium	8 (35)	
High	6 (26)	
Unknown		
Health literacy (HL) level		
Lower score SBSQ^a^	7 (30)	
Higher score SBSQ^a^	14 (74)	
Unknown score SBSQ^a^	2 (9)	
Lower score NVS‐D^b^	4 (17)	
Higher score NVS‐D^b^	9 (39)	
Unknown score NVS‐D^b^	10 (43)	

^a^ For the SBSQ, individuals with one or more answers not indicating the highest Hl category were considered lower HL (mostly: reading and understanding) skills. Note that we used a different cut‐off point compared to the original study.

^b^ For the NVS‐D, individuals with < 4 out of 6 correct responses were considered lower HL (mostly: calculating/numeracy) skills.

BOX 2 **Decision situations that occurred during the consultations.**

‐ Whether or not to continue with disease‐modifying treatment.
‐ Switching to another type of disease‐modifying treatment (e.g., from Natalizumab to Ocrelizumab).
‐ Whether or not to start with physiotherapy/outpatient rehabilitation.
‐ Whether or not to continue with physiotherapy/outpatient rehabilitation.
‐ Whether or not to treat pain actively (e.g., use of [a combination of] pain medication, psychological support, referral to hospital ‘pain department’).
‐ Whether or not to refer for a psychological diagnosis (e.g., depression).
‐ Frequency of MRI scan (with stable MS): Less often or continue with the current frequency.
‐ Frequency of MRI scan (with uncertainty about MS progression): More often or continue with the current frequency.
‐ Whether or not to refer to neuropsychological examinations (SOMSCOG poli).
‐ Whether or not to refer to dermatology.
‐ Whether or not to refer to urology.
‐ Whether or not to refer to.
‐ Whether or not to undergo treatment abroad.

We assessed the NVS‐D among 13 patients and four of them were identified as lower health literate. The SBSQ was administered among 21 out of 23 patients. When we followed the cut‐off (an average score of ≤ 2 indicated inadequate health literacy) as described by Chew et al. [37], no one was classified with inadequate health literacy. Because of potential socially desirable answers of patients—since the HCPs screened patients with the SBSQ—we adopted a less strict cut‐off: Patients not providing the most optimal answers to one or more of the three questions were classified as ‘lower’ health literate. Using this cut‐off, a total of seven patients had ‘lower’ health literacy. The four patients who were classified as lower health literate using the NVS‐D were also classified as such using the SBSQ. A total of 17 patients and seven HCPs were included for an interview.

### 3.1 Themes Derived From the Consultations and Interviews

A total of five main themes with different subthemes were identified in the data analysis, which are described in Tables 2, 3, 4, 5, 6 with illustrative fragments from consultations and interview quotes. The quotes were translated into English by the first author and reviewed by the other authors. The first four themes were related to how HCPs used diverse types of outcome information at the individual (*N* = 1) and aggregated (group) levels in clinical encounters, and which steps/elements of SDM were thereby supported (RQ1). The fifth theme describes the overall differences between lower health literate patients versus higher health literate patients (RQ2).

**Table 2:** Theme 1 with illustrative quotes.

Theme/subtheme	Illustrative quotes from consultations	Illustrative quotes from interviews with HCPs	Illustrative quotes from interviews with patients
**Theme 1: Use of outcome information mainly for monitoring and management**			
1a: HCPs focused on giving and explaining a good clinical treatment advice	Consultation with patient 6, male, 46 years, medium educational level, higher health literacy Clinician: ‘Did you already see the MRI results?’ Patient: ‘I saw it. It wasn't really in a language I could understand, but what I saw in the conclusion was “an unchanged image”, so yes, that tells me the most’. Clinician: ‘And when I look back at those MRIs now, there has actually been no change at all for 2.5 years, more than 2.5 years. So that means that there are no new lesions in your brain and that also means that, yes actually, it is stable in itself and that is of course very good'. Consultation with patient 9, male, 31 years, higher educational level, higher health literacy Clinician: ‘We mainly look at all those scars to see if anything has occurred. As an indication of whether something happened somewhere in that year. And that's actually not the case, so that looks very calm’. Patient: ‘Fortunately it's positive’. Clinician: ‘Yes. Certainly. So for me, I would say we'll continue like this’.	Clinician 1, neurologist For me, if the MRI scan is stable or not stable, that will influence my advice whether or not to switch*.* Clinician 8, rehabilitation clinician ‘Because I say “well, we as a team have discussed that”, right, when you talk about rehabilitation. We see that it is useful to continue in a different way’. Well, and I also get that information from my physical examination. Because based on my physical examination—clinical outcomes I should perhaps say—you also determine if there is indeed a choice for this patient and what the choice is.	Patient 6, male, 46 years, medium educational level, higher health literacy Interviewer: ‘What information has stuck with you?’ Patient: ‘That it's stable’. Interviewer: ‘That was the MRI result, right?’ Patient: ‘Right. Yes, and she suggested maybe doing the MRI every two years, but then I said I'd rather just do it annually and that was also fine’. Patient 13, female, 60 years medium educational level, lower health literacy ‘During the MRI scan I am always curious about where the spots are. He says “There is a very small spot in your head, on the left lobe, very small”. And then he also said‐ and he had also said that before‐ “if you continue with those medications, we hope that spot will go away”’.
1b: Limited/modest added value seen by HCP for using PROMs	Consultation with patient 20, male, 52 years, higher educational level, higher health literacy Clinician (referring to individual PROMs results): ‘And I saw that you also indicated some problems with sexual functions and that you would like help with that. Shall I you tell you something more about that?’ Patient: ‘Yes, I thought about that before. I think it works sexually, and it's still just nice. But yes, I do have the feeling that my erection is a bit less, and I have thought about it before, of course there are remedies for that. What is that called again?’ Clinician: ‘Viagra’. Patient: ‘Yes, exactly, Viagra‐like drugs. I would actually like to have such a remedy, if possible’. Consultation with patient 8, male, 48 years, higher educational level, higher health literacy Clinician (after discussing the stable MRI result, not referring to individual PROMs results): ‘How has the rest of the year been in terms of MS or other things?’ Patient: ‘Well, calm’. Clinician: ‘Have there been any changes?’ Patient: ‘Well, sometimes, I still have it in my feet, that it is a bit numb, such a numb feeling’.	Clinician 4, neurologist When the patient tells me that walking is difficult. Yes, then I don't really need a walking test or a PROM to have a conversation about that. Clinician 2, neurologist ‘Just ask how you are doing, how are you walking, how are you urinating, are you tired, how are you doing at work, are you satisfied with your medication … And that is also in the PROMs, which I don't use much, because I think it's a lot, because I just forget, because I'm very used to doing it the old‐fashioned way'. Clinician 8, rehabilitation clinician 'It is a nice ‘peg’ to hang things on. When I see in the questionnaire that the patient suffers a lot from fatigue and, well, sometimes I can include related topics. [..] I think it's a really easy way for a patient to think in a quiet moment about what his or her request for help is and what areas he or she has problems with’.	Patient 20, male, 52 years, higher educational level, higher health literacy 'Because by using this questionnaire, with the questions too—because sexuality was included in it. I think oh yes, that works out great. Then I fill that in. [..] Yes, because without the questionnaire I don't think I would have discussed that topic with her. And maybe I wouldn't have thought about it either’*.* Patient 23, female, 55 years, lower educational level, higher health literacy Interviewer: ‘Do you also remember if you completed a questionnaire before the consultation?’ Patient: ‘Yes, I did that in fact.[..] it was indeed about symptoms of MS and how it indeed affects your daily life. Yes, I quickly participate, because I want to contribute to making it better for the future’.

**Table 3:** Theme 2 with illustrative quotes.

Theme/subtheme	Illustrative quotes from consultations	Illustrative quotes from interviews with HCPs	Illustrative quotes from interviews with patients
**Theme 2: Choice cautiously and implicitly presented to patients**			
2a: No raising of choice awareness	Consultation with patient 1, female, 45 years, educational level unknown, higher health literacy Clinician: ‘But especially if you notice that if something is too much for you, that it causes an increase in complaints, then it is now a good idea to look at it or perhaps ask for help with it. I don't know if you've considered that?’ Consultation with patient 15, female, 53 years, lower educational level, lower health literacy. Clinician: ‘So sometimes some difficulty urinating’. Patient: ‘Yes’. Clinician: ‘Is that something else that you say: it bothers me quite a lot, because sometimes you can use medication or physiotherapy or things to improve it. Is that something that needs to be looked into or are you like: it's okay for now?’ Patient: ‘I'm like, just leave it for a while, because I already have two pills for peeing. Let's wait until everything calms down a bit, because there is a lot of stress around me’.	Clinician 2, neurologist ‘I think what goes well is that at some point I say, hey, so we have to think about this or that. Maybe I don't call it that yet … I think it's very logical that you have to make a decision about that. I don't know if the patient finds that as logical as I do. So maybe that could be made even clearer'. Clinician 7, rehabilitation clinician ‘If a patient indicates “I have increasing problems with my hands or feet, I no longer walk well”, or whatever, then I investigate that. And then I start thinking about if I also see this in the physical examination. And well, then the question is: are there treatment options for this? Well, you discuss that with the patient and the pros and cons of it’.	Patient 22, female, 49 years, higher educational level, lower health literacy ‘No, she just said on that basis: “I'll send an email to the GP and the physio”. So basically it sounded ready‐made, really’. Patient 10, female, 28 years, higher educational level, higher health literacy Yes, she took me by surprise a bit with that. That she wanted me to start using new medication?
2b: Patients wanted to hold on to what went well	Consultation with patient 4, female, 55 years, medium educational level, lower health literacy Clinician: ‘You have been very stable for a long time using this medication, so that's going well. Or did you start to think differently about Natalizumab?’ Patient: ‘No, that's fine. It's going well, really well’. Clinician: ‘Are you still happy with it?’ Patient: 'Yes, I am satisfied with that. Sometimes I think I maybe I could go only once every six months with the other medication’. Clinician: ‘That Ocrelizumab?’ Patient: ‘Yes, I sometimes think about it, but I also think, it's going well now, I should just leave it as it is’. Consultation patient 5, female, 21 years, medium educational level, higher health literacy. Clinician: ‘Yes, and for now I don't really have much else’. Patient: ‘Yes, actually neither do I. It's going so well, so that's nice’. Consultation with patient 10, female, 28 years, higher educational level, higher health literacy. Patient (after discussing another medication option): ‘Well, I'm going to keep that in mind. I'll think about it. Yes, I now think that I'm doing well'.	Clinician 7, rehabilitation clinician And anyway, yes, if a patient is still in a certain process of processing, for example his diagnosis at all, then I have the feeling that they are not actually ready for that yet too. That they are not sufficiently open to actually see what the different choices are and their consequences. I find that difficult. Clinician 3, neurologist. 'Sometimes I see people with a lot of uncertainty at the beginning, and of course, you don't know what your life is going to look like and you are already a bit topsy‐turvy about it. And then I notice that when the conversation turns to that, it can sometimes become more emotional. And sometimes I see people who don't ask questions and then my guess is: maybe they don't dare to ask that question because they don't dare to hear the answer’.	Patient 4, female, 55 years, medium educational level, lower health literacy Well, we briefly talked about that medication. And that other medication that you get once every six months. That actually seemed like something to me. But, I've been going every eight weeks now, so I also thought: yes, this just works well for me. And so we just left it at that. Patient 19, male, 50 years, higher educational level, higher health literacy That has to do with the fact that I simply have the fear of deteriorating quickly again. Patient 6, male, 46 years, medium educational level, higher health literacy Now things are going just fine so I don't need anything else.
2c: Limited option talk nor use of aggregated outcome data, while patients did want it	Consultation with patient 6, male, 46 years, medium educational level, higher health literacy Clinician: ‘There's one thing I want to discuss with you. We have been scanning regularly since 2018. Now there has been nothing for 2.5 years, you feel good too: do you need to continue scanning annually or do you say “let's expand that”? Because if you ask me, we can also expand it, that we say “we won't do it for a year”. Because imagine that next year there will be a very small spot, then I wouldn't say we need to treat that. But if you say “I really like having that feedback” then we can continue’. Patient: ‘Yes, to be honest, I like to do it every year. I find it exciting, I have to be honest, but it is nice to see afterwards’. Consultation with patient 8, male, 48 years, higher educational level, higher health literacy Patient (after informing about options himself): ‘But if it's still stable next year, maybe we can talk about tapering off medication?’ Clinician: ‘Yes. We can talk about that for how long will you continue with medication or not? And how useful is that? We partly don't know that. Because well what would be the course of your disease with or without medication? Of course, we don't have a copy of the same person walking around without medication. But it is certainly open to discussion’.	Clinician 1, neurologist 'That I have not used it [option grid] myself so far because yes, I feel that it is not yet officially approved, that is because the guideline is currently being revised, so this is partly not up to date. But my own thought is that it is especially useful for a consultation with a neurologist or a nurse who is less informed about all MS treatment options and yes, I have that in mind, but it is true that I may be depriving patients of the opportunity to read it carefully again’*.* Clinician 4, neurologist 'What I think could be improved is that, for example, if you look at aggregated results, information about trials and such, that sometimes there is so much that it is almost impossible to keep up, so to speak, for yourself as an individual'. Clinician 5, rehabilitation clinician You may think about choices between different types of treatment. Or implementation of treatment. So where that takes place and with whom Staging of the treatment. Those are usually the options available.	Patient 4, female, 55 years, medium educational level, lower health literacy (After a probe from the interviewer): ‘No I haven't actually heard any pros or cons about that psychological research’. Patient 6, male, 46 years, medium educational level, higher health literacy Interviewer: ‘And did she also explain the benefits and harms, in your opinion? Related to, annual scanning or just’. Patient: ‘No, not. [..] I didn't understand that there were benefits and harms I did not understand that’. Patient 8, male, 48 years, higher educational level, higher health literacy But then it would have been useful if various alternatives were also presented. Indeed, well, things are going well now, things are going stable, we could think about, these are the options, these are the pros and cons.[..] if there are different alternatives then the doctor will know what's best. But it is still good to know why this is chosen.

**Table 4:** Theme 3 with illustrative quotes.

Theme/subtheme	Illustrative quotes from consultations	Illustrative quotes from interviews with HCPs	Illustrative quotes from interviews with patients
**Theme 3: Active use of information on patients' experiences and priorities in treatment advice**			
3a: HCP related the experiences of patients to individual outcome information and treatment advice	Consultation with patient 10, female, 28 years, higher educational level, higher health literacy Clinician: ‘And in terms of your complaints, it is quiet, but on the MRI scan, yes, there is some disturbance every now and then, not very pronounced, but something. In any case, it is a good idea to discuss together whether you would like to switch to a second‐line drug?’ Consultation with patient 17, female, 70 years, medium educational level, health literacy unknown Patient: ‘And now my fear is that it is because of that burden, that I may have a relapse. And yes, how do you see that?’ Clinician: 'Actually, more about how you can best continue your recovery, that is maybe your underlying question? And are there specific things to take into account with the background from the MS?’	Clinician 3, neurologist What is also important is that there are certain treatments that sometimes involve some side‐effects or other things and people have to keep up with that. Clinician 1, neurologist The MS disease course and that there is uncertainty about it, I also consciously point out that that is very logical and very normal and that, if there are certain complaints of which one is not sure if it's MS‐related, gosh, please bring it up. So that is in any case discussed. So that's a regular part of my conversations.	Patient 22, female, 49 years, higher educational level, lower health literacy It was actually just a conversation with a lot of questions and how I experienced everything. Patient 13, female, 60 years medium educational level, lower health literacy About Fampyra, we talked about how those medications didn't work, it wasn't good … Yes, it was good, but not good for me. I had a lot of headaches that made me feel unwell.
3b: HCPs gave treatment advice and patients quite passively consented	Consultation with patient 2, female, 57 years, unknown educational level, lower health literacy Clinician: ‘Because my plan is to at least continue with the Gilenya, because in any case we see on the MRI scan that it is calm now and of course that was different last time And we also consider it important to have a skin inspection done once in a while when people use Gilenya, because this sometimes makes the skin a little more restless’. Consultation with patient 9, male, 31 years, higher educational level, higher health literacy Clinician: ‘Does it bother you?’ Patient: ‘No. Not at all. [..] But I have now started taking it in the evening, because otherwise it would make me feel nauseous. But the only thing I notice now is that I'm a bit more tired and a little bit of stomach pain, but nothing else’. Clinician: ‘Is it bearable?’ Patient: ‘Yes, because I'm just working’. Clinician: ‘Shall we continue like this?’ Patient: ‘Great’. Consultation with patient 10, female, 28 years, higher educational level, higher health literacy Clinician: ‘And then I think yes, if we will switch because you're not able to adhere to it all the time, then I would be inclined to switch to such an infusion. So take that into account’. Patient: ‘OK, OK’.	Clinician 5, rehabilitation clinician I usually give advice and think, well, that's what I would do in this case. Then I leave it up to the patient to ultimately determine the choice. Clinician 2, neurologist. ‘So then I make a decision in advance about what I think is good for suppressing the MS, but what is also good for the patient and I think: what will best help this patient?'	Patient 22, female, 49 years, higher educational level, lower health literacy ‘Do I think that there was space for that, yes. I didn't take it, but I think there was room for that, yes. She did ask if that <advice> was okay, so I think I said that it was fine with me’. Interviewer: ‘Yes, that was indeed the case. Would you have liked to have had more input into this, in addition to giving consent?’ Patient: ‘Well, I think I … Yes, I certainly felt like I had more input on that … needed that’. Patient 20, male, 52 years, higher educational level, higher health literacy ‘That was the appointment with a sexologist or getting a drug indeed. And my preference was actually getting the drug. But doctor Y indicated that she preferred the appointment. But I did feel she gave me freedom in that. So yes, I felt like I was free to do so, but her advice was to make an appointment. I guess, well, I just found it helpful to follow that advice. [..] I think yes, if she advises to do it that way, then I will'.

**Table 5:** Theme 4 with illustrative quotes.

Theme/subtheme	Illustrative quotes from consultations	Illustrative quotes from interviews with HCPs	Illustrative quotes from interviews with patients
**Theme 4: Role of smart information technology**			
	Consultation patient 5, female, 21 years, medium educational level, higher health literacy Clinician: ‘Yes, exactly. So the doctor from the multidisciplinary consultation will say tomorrow whether she still wants another injection. Um, and the next Natalizumab treatment is scheduled for June 2. And then an appointment for the MRI on June 15, and then on the 28th for me’.	Clinician 1, neurologist While if you have good PROMs system ‐I also think there are more of a kind of decision support providers‐ in which a neutral explanation is given of what is important to know in relation to those medications. Suppose that we don't ask as doctors and patients don't know that it is can play a role, then it will not be discussed. Clinician 2, neurologist ‘A clear drawback is that you really have to click through a lot and you have to read quite a lot, I think, what the patient fills in [..] What would make it better is some kind of highlights of “these are the things you should really do something with”. So, hey, only things that are really permanent or “this is what the patient wants to talk about”, because that's actually what's most important to me’.	Patient 14, female, 48 years old, higher educational level, lower health literacy, possibly related to cognition problems ‘Especially during conversations, I start to doubt myself about if I understood it correctly and then its more pleasant to be able to read it again. I can't remember it all’. (After probe interviewer): ‘I think I'll write a summary on paper so you can read it yourself’. Patient 22, female, 49 years, higher educational level, lower health literacy I think it's always important that one receives some kind of summary afterwards, by letter or email, or whatever, so that people can read it again at their own pace.

**Table 6:** Theme 5 with illustrative quotes.

Theme/subtheme	Illustrative quotes from consultations	Illustrative quotes from interviews with HCPs	Illustrative quotes from interviews with patients
**Theme 5: Less outcome information, less SDM among lower health literate patients**			
5a: Classical information–related problems	Consultation with patient 9, male, 31 years, higher educational level, higher health literacy Patient: ‘It was indeed about the blood values and the X‐ray results, but everything else was fine, I think, except that the liver values were somewhat elevated'. Clinician: ‘Yes. You had already checked those yourself, the values?’ Patient: ‘Yes. I often do that. When I get a notification from the MyChart app, I always check it myself’. Consultation with patient 14, female, 48 years old, higher educational level, lower health literacy, possibly related to cognition problems Clinician: ‘Are there any other things you would like to discuss?’ Patient: ‘Yeah, well. X is also listening and I just don't really know how to respond to everything. I will do my utmost to do it well’. Clinician: ‘Yes, indeed. But you have to say if things are going too fast or are unclear’. Consultation with patient 13, female, 60 years, medium educational level, lower health literacy Clinician: ‘It is important to correct the perception that the siponimod, ‐even if it would work very well and without side effects‐ that it would make you walk any better. What it does is trying to stop deterioration. To ensure that you do not go further backwards’. Patient: ‘I don't want to get worse because I think that's very bad. I also don't feel like getting a relapse anymore’. Clinician: ‘No, and that's what Siponimod is actually intended for, namely if you calm down the disease, calm down the inflammation, then you hope that you will not have another attack. Then you hope that you don't go further backwards. But that is different from getting better Because Siponimod actually can't do that’. Patient: ‘No, yes, I don't know. Is the medication still useful? I want to know that from you’.	Clinician 2, neurologist ‘I notice that I take more of the lead with these [lower health literate] patients. These are quite complicated decisions about what type of medication. I try to ask them what is important to them. But that is also a quite difficult question, isn't it? [..]. So I take more of the lead in that I say, “Hey, this seems like a good idea to me, do you think so too?” So then I fill it in a little more, because I think that person needs more guidance’. Clinician 8, rehabilitation clinician It sometimes helps to not only talk but also to give something visually, such as making a drawing or writing it down. I think I should write it down more often because I actually don't make drawings enough, I would actually like to do a little more, it just often supports your story and not everyone can read that well or understand written or spoken words. Clinician 4, neurologist Patients who, for example, have very uninformed, very clear beliefs, are more complicated. Yes, and it also falls under limited health literacy, because yes, limited health literacy does not mean that you do not have an opinion, so to speak. Sometimes people have very strong opinions, but they actually want the wrong thing for themselves.	Patient 20, male, 52 years, higher educational level, higher health literacy Yes, you could get a certain brain disease. Then you can just die. Yes, I was a bit shocked by that. Then I also delved into figures, international results, research, science, pieced together all kinds of things. And then that chance actually turned out to be very small Just one in thousands or even higher [the denominator]. And that these were really people who also had all kinds of underlying things. Then I thought well okay and based on that I made my choice. Patient 9, male, 31 years, higher educational level, higher health literacy Sometimes I do have questions in advance, a list that is So I write it down otherwise I'll forget it. Patient 13, female, 60 years, medium educational level, lower health literacy I usually take someone with me. I prefer to take my daughter and my ex‐husband always came along. And they all remembered it well. [..] My daughter is very smart and she remembers everything. Mama, they said that, said that. I think, oh I didn't hear that at all. That passed me by, shame. […] Or then I think I'll have to remember a word, like Fampyra, it is now very clear in my head, I really have to train to remember that. Patient 11, female, 41 years, medium educational level, health literacy unknown but shows interpretation problems in the consultation) A lot has been discussed. A lot and I can't even remember most of it.
5b: Lacking choice/SDM awareness	Consultation with patient 13, female, 60 years, medium educational level, lower health literacy Clinician: ‘You could also say we stop it and do a new MRI scan in six months to see if there's still activity there or not? And then we can use that MRI scan to discuss again if you want to try again if the medication works or you can say I don't want it and then we can decide partly based on the situation of that MRI scan, that says how active is the disease?’ Patient: ‘Yes, this morning. I said to a woman: “It seems like it is working because I now feel that I am not so dizzy”. Because it's usually early in the morning when I'm sitting in bed. I think Oh, I have to hold my bed all the way. I'm terrified of falling out of that bed. So then I really have a very dizzy state and I think oh my gosh, is it starting again?’ Consultation with patient 22, female, 49 years, higher educational level, lower health literacy Clinician: ‘How could we help you with the left leg, what do you think you need?’ Patient: ‘I have no idea. We talked about it and I was referred. I think: okay, what do they want?’	Clinician 1, neurologist ‘But I think especially with people with a different cultural background or a language barrier, there are certainly some people there. I also have a few patients with lower intelligence or simple‐minded or whatever you want to call it [..], Then I am more directive, for example “I think that in your circumstance I would advise this or that”’. Clinician 2, neurologist. So where other people might want to hear four or five options, I often say one option. And then I say: how does that sound, for example, because there are other options? And then I can name them briefly, but then I keep it shorter and more concise and I try to keep it easier. So just less information actually. Clinician 4, neurologist ‘Yes, the easiest case is the patient who says “I don't know and I don't understand it anyway and I trust that you can make the best decision for me”’.	Patient 4, female, 55 years, medium educational level, lower health literacy I never feel like I have to participate in the decision about this or that or so. Look, of course you're going to ask some things sometimes, like: well, maybe that could be done or that could be done, in case there is something particular. But not like: well, I eh have to participate in the decision. Patient 11, female, 41 years, medium educational level, health literacy (unknown but shows interpretation problems in the consultation) You can't really say anything about that. Yes. Only whether it is suitable or not. I can't say more about it.
5c: Other types of affective (coping) reactions’	Consultation with patient 12, female, 37 years, unknown educational level, higher health literacy Patient: ‘Because I was also a bit worried about those blood values.[..] There is a little voice in your head somewhere that is sometimes concerned with it, like: oh, values are bad, soon I will be that one out of a million. Yeah, that's not a good feeling either’. Consultation with patient 15, female, 53 years, low educational level, lower health literacy Patient: ‘Then I got the results of that blood test and the liver values had tripled. I was quite shocked by that’.		Patient 9, male, 31 ears, higher educational level, higher health literacy. Well, then uncertainty, I think it was more difficult for me to deal with it then, that I had this, and yes, I found it more difficult the disease itself.[..] And I just knew less about it. By now, well, I've read a lot and had a lot of conversations. Patient 13, female, 60 years, medium educational level, lower health literacy. Look, someone called me about that medicine. You can get this, that entire list of everything I got or could get. I think okay, I'll see. Patient 22, female, 49 years, higher educational level, lower health literacy And then I like it when someone reassures you, but doesn't dismiss it as ‘something they hear often, but it's nothing’. That really bothers me, then I get … I do get angry, but I'm not going to get angry over the phone, so to speak. Then I'm like: 'okay, you see, never mind, I'll never call anyway, fine. And then I'll solve it myself’. Interviewer: ‘Okay, so how do you solve that?’ Patient: ‘Then I won't think about it anymore’.

#### 3.1.1 Theme 1: ‘Use of Outcome Information Mainly for Monitoring and Management’

In the consultations, HCPs mainly used outcome information to monitor patients' health status, and this mostly concerned clinical outcome information at an individual level, such as an MRI result or blood values.

Underlying this main theme was subtheme 1a: ‘HCPs focused on giving and explaining a good clinical treatment advice’. HCPs typically used individual outcome information, such as an MRI result, to monitor the effect of treatment or non‐treatment. They extensively explained the meaning of this information, mostly in terms of the progression of MS and the influence of treatment. In the interviews, HCPs indicated that they mainly used outcome information this way, to give direction to the conversation and to proceed to giving good treatment advice. Patients in the interviews acknowledged those extensive explanations; they said that they were worried about MS progression and that they needed information about the relation between complaints and MS. They also typically remembered this clinical information and the interpretation given by HCPs in terms of the MS being stable or not.

Subtheme 1b was ‘Limited/modest value seen by HCP for using PROMs’. Although a few HCPs did discuss PROMs results in consultations, most were not unequivocally positive—although neither explicitly negative—about using PROMs for monitoring and management. This seemed to be a general attitude towards using PROMs, and not specifically towards the PROMs used in their academic centre. Some HCPs found the current PROMs used in the academic centre not user‐friendly, but most HCPs said that not using PROMs was more a question of inattentiveness or that it did not add value above their routine in asking questions to patients. Patients indicated in interviews that they considered the impact of MS on their daily lives essential to discuss with their HCP and that they were willing to complete PROMs about this, although they did not immediately see the benefit if HCPs would not feedback on the results.

#### 3.1.2 Theme 2: ‘Choice Cautiously and Implicitly Presented to Patients’

Despite the fact that multiple ‘smaller’, and some ‘bigger’, decision situations occurred during consultations as a result of discussing issues or problems (Box 2), HCPs only cautiously and implicitly presented choices to patients. This may be related to the type of consultations observed, which were focused on care management and not on the potentially more clearly defined first‐time decision about DMT.

Subtheme 2a was ‘no raising of choice awareness'. HCPs did not explicitly refer to the fact that a decision was to be made, nor to the fact that the patient's voice was important in this. In those cases that choice was discussed, this mostly occurred after discussing a patient's complaint, for example, in case of a patient's sexual complaints, one neurologist said: ‘You could consider a visit to a sexologist’. In the interviews, HCPs said that treatment plans discussed in the consultations were not typically definitive and that there is always room to revisit decisions if needed. When we asked patients in the interviews about the choices offered, they generally did not remember this. Most said to remember SDM around their initial decision about using DMT, although no one could remember the exact benefits and harms of options discussed at that moment in time.

Subtheme 2b was ‘patients wanted to hold on to what went well’. In the interviews, patients indicated that they wanted to hold on to what went well, either using medication or other treatment, and that ‘doing nothing’ was not really an option for them. The type of consultations observed might play a role in this because these focused on care management after the decision about DMT. When HCPs did present choice in some way—often implicitly—patients typically indicated that they wanted to stick to the previously chosen treatment plan (e.g., ‘I'll leave it for now’).

Subtheme 2c was ‘no option talk nor use of aggregated outcome data, while patients did want it’. Probably as a result of themes 2a (no raising of choice awareness) and 2b (patients holding on to what went well), we hardly observed the discussion of options. Likewise, the use of aggregated outcome data, for example, from clinical guidelines or presented in PtDAs, was not seen. When choice was discussed, the options were briefly mentioned by HCPs, with a focus on the option preferred by the HCP. The options discussed were mostly DMT options, and HCPs explained to patients what those options generally involve (e.g., pill vs. drip) and no further benefits and harms. Probability information was hardly provided and only in verbal terms. In the interviews, patients indicated that they did need transparent information about options available, and also when they were satisfied with their current treatment plan. When we asked HCPs about this part of SDM in the interviews, they said that they sometimes use aggregated outcome data to give information about options, but that it was difficult for them to have immediate access to up‐to‐date information.

#### 3.1.3 Theme 3: 'Active Use of Information on Patients' Experiences and Priorities in Treatment Advice’

We observed HCPs' active exploration and discussion of patients' experiences, priorities and daily context. Nevertheless, we did not see this being translated to deliberation, since HCPs typically proceeded quickly to provide treatment advice (see Theme 2).

Subtheme 3a was ‘HCPs related the experiences of patients to individual outcome information’. HCPs actively inferred about patients' experiences in daily life, both concerning living with MS and the use of DMT or any other treatment. In doing so, HCPs not only inferred patient priorities (e.g., ‘What bothers you most?’) but also related those experiences to previously discussed individual outcome information (Theme 1).

The other subtheme 3b was ‘HCPs gave treatment advice and patients quite passively consented’. After discussing patients' values and priorities, HCPs appeared to quickly proceed to providing explicit treatment advice. This advice was often to proceed with the chosen treatment, now and then with ‘small’ changes to the plan, such as undergoing an extra MRI or decreasing/increasing the medication dose. In several consultations, treatment advice was given less explicitly and more in a question form, such as ‘So let's go on like this?’. Patients quite passively agreed with advice given, especially when the MS was fairly stable. They generally did not ask questions about other options nor about the benefits and harms of changing treatment plans. In the interviews, patients noticed that they tended to consent to the advice and that they did not want too much change, ‘as long as it goes well’ (subtheme 2b).

#### 3.1.4 Theme 4: ‘Role of Smart Information Technology’

This theme concerned that smart technology might be needed to utilize outcome information more explicitly during consultations. We observed that relatively much time in the consultations was spent to information about practical issues. For example, when the HCP and patient agreed on the way to proceed, they discussed what is needed to arrange an extra lab test, an MRI or a referral to another clinical speciality. Patients were sometimes checking if they had understood this information correctly. In the interviews, patients expressed a need for a summary of the consultation with individualized information, preferably with visuals. Interviewed HCPs indicated to welcome more smart organization of PROMs in electronic patient files, as well as guidance or templates to integrate PROMs information with their questions to patients in consultations. Some HCPs also suggested that outcome information at an individual level could be more explicitly used to raise choice awareness. Patients, in turn, said that they valued different types of outcome information, but that they needed help in interpretation and ideally receive summaries of key information.

#### 3.1.5 Theme 5: ‘Less Outcome Information, Less SDM Among Lower Health Literate Patients’

In the consultations with patients classified as lower health literate, we observed the use of outcome information as well as SDM to a lesser extent compared to consultations with higher health literate patients. When the HCPs discussed outcome information with lower health literate patients, they generally did so in the same manner as with patients with higher health literacy, although they did seem to put less emphasis on explaining the meaning of outcomes (see Theme 1a).

Subtheme 5a was ‘classical information‐related problems’, which meant that lower health literate patients seemed to experience typical information‐related problems known from the literature. In the consultations, some of those patients seemed to misinterpret the information provided to them, for example, about the expected benefit of medication. HPCs confirmed this in interviews afterwards. Moreover, in the consultations, patients with lower health literacy asked fewer questions compared to those with higher health literacy. Lower health literate patients themselves indicated, both in the consultations and in the interviews afterwards, that they did not actively prepare consultations, rather not look up information in their electronic patient file and sometimes have difficulty following exactly everything being said.

Subtheme 5b was ‘lacking choice/SDM awareness’. In the consultations with lower health literate patients, the notion of ‘choice’ appeared to be even more absent, compared with higher health literate patients. This seemed to stem from both parties: HCPs indicated that they wanted to take more guidance in encounters with these patients, and lower health literate patients seemed to have less notion of choice and the associated available options. Even if HCPs did offer some form of choice in the consultations, lower health literate patients hardly responded to this. Some lower health literate patients just proceeded with telling their story, for example, about their complaints, instead of responding to the choice offered.

Subtheme 5c was ‘other types of affective (coping) reactions’. In the consultations, we observed that lower health literate patients occasionally responded ‘shocked’ by results, for example, from MRIs or blood tests. HCPs often responded by reassuring these patients, and by giving realistic but positive interpretations to the outcome information. In contrast to higher health literate patients, who often expressed concerns and feelings of uncertainty, lower health literate patients did not express any of such feelings in the consultations nor in the interviews. This may explain why HCPs also put less emphasis on explaining outcome information in detail. Higher health literate patients tended to emphasize more in the interviews how they coped with their concerns and uncertainties, for example, by talking to others about it and by searching for additional information.

## 4 Discussion

### 4.1 Discussion of Findings

This study aimed to investigate how SDM and the use of different types of outcome information are applied in routine care management for people with MS in an academic outpatient clinic. The 23 observed consultations included several ‘smaller’ consecutive decisions after the first time ‘big’ decision about the use of DMT. For example, patients and clinicians talked about ways to deal with physical complaints and, in case of absence of complaints, the frequency of diagnostic MRI testing. We observed little use of outcome information, apart from the more classical clinical outcome information at an individual level such as MRI or lab results. This clinical outcome information was mainly used for monitoring and management purposes, and its use did not automatically lead to a process of SDM. HCPs overall cautiously and implicitly presented choices to patients, and especially took control in decision‐making with patients with lower health literacy. In the interviews, patients indicated that they tended to consent to the advice given by HCPs and that they preferred not too much change in treatment plans, as long as things went well. However, they also said they wanted to be informed about the available options with their benefits and harms.

The emphasis in the observed consultations was placed on care management after the ‘big’ decision about DMT and after an initial treatment plan had been made. In light of this, it makes sense that HCPs focused more on discussing patients' experiences and priorities than on all available options with benefits and harms. However, in the interviews, HCPs indicated that treatment plans can be revisited if needed, and patients said they wanted to know the options available to them, also in case they did not immediately want to change their treatment plan. We observed several ‘decision situations’ in the consultations, such as continuing or switching DMT, but also, for example, the choice to be referred to another medical speciality or to start physiotherapy in case of physical complaints. However, HCPs presented these choices rather cautiously and implicitly, which may have made it hard for patients to recognize this as a choice. For patients with lower health literacy in our study, this even seemed more problematic, in line with previous literature [30]. It should be noted that there had been no interventions to implement SDM on the department in our academic centre. Although some HCPs indicated to have received some SDM training in the past, there had been no training in this specific department. This may explain for a great part why the step of creating choice awareness was largely absent in the consultations. A review of studies using the OPTION instrument—an SDM observing instrument—also showed that without interventions to implement SDM, HCPs generally do not demonstrate that they try to involve patients in decisions [40].

We observed active discussion of patients' experiences and priorities. Several studies have concluded that this is the step that may need the most improvement in practice [40, 41]. HCPs in MS care did not seem to have much difficulty implementing this part of SDM, but it should be said that HCPs themselves did not relate this part of their clinical encounter to offering choices and sharing decisions about the treatment plan. So, it seemed more their ‘natural way of working’ than a result of training. Specifically for a condition such as MS, this may be explained by the fact that HCPs have a longer term relationship with their patients, and probably knowing relatively much of patients' daily context. In training HCPs more fully in SDM, it might be fruitful to relate HCPs' apparent natural way of discussing patient priorities to key decision moments with benefit/harm information about the options available.

PROMs were not actively used in consultations with patients, despite the fact that HCPs were not explicitly negative about the use of PROMs. Patients said that they were also willing to fill in PROMs, but that they would like HCPs to feedback on the individual results in a consultation. On the few occasions that HCPs did feedback on individual PROMs results, such as fatigue or urinary problems, a tentative discussion emerged about the options to deal with that particular problem. The lack of use of PROMs may thus indicate a missed opportunity for SDM. Our findings seem to resonate with the findings of van der Willik et al. [42], who reported a need among HCPs for ‘guidance on how to act on PROM results and those of Westerink et al. [17], who reported that HCPs found it ‘challenging to translate outcome data into treatment decisions’. The use of PROMs and SDM are often mentioned in the same breath, but no expert consensus seems to exist yet on their collaborative utilization [8]. Training in this field may focus more on this integrated application, for example, in role playing in which HCPs use PROMs to signal ‘problems’ and more explicitly mention choice and the options to deal with the problem.

### 4.2 Study Strengths and Limitations

A strength of this study is that we combined interview data with observational data. Although there is an increasing interest in the use of multiple types of outcome data for SDM, most studies have relied on interviews with HCPs and patients. Our study adds to this literature by observing what is happening in clinical practice. By combining observations in practice with the opinions and perceptions of patients and HCPs, we were able to deepen our understanding. We acknowledge that our study was performed in an academic hospital setting and one of the few MS centres in the Netherlands; findings may not be representative of other Dutch hospital settings in which routine care is provided to patients with MS. We also acknowledge that the group of people with lower health literacy was relatively small and that we cannot generalize these findings. Our data collection took place in 2022, not long after the COVID‐19 pandemic. Findings should be interpreted because patients with MS might have felt very vulnerable during or right after the pandemic.

## 5 Conclusions

Overall, SDM and the use of different types of outcome information did not seem to be enacted in routine care management for people with MS, mostly because no explicit choice awareness was created. However, we did find a useful natural attitude among HCPs towards the elicitation of patients' priorities and experiences. Further training in the collaborative use of PROMs and SDM seems a reasonable next step to proceed in providing patient‐centred care to people with MS. Furthermore, patient information about the key decision moments with links to benefit/harm information about options available seems needed to fulfil patients' need for information.

## Author Contributions

**Olga C. Damman:** conceptualization, investigation, funding acquisition, writing–original draft, methodology, validation, formal analysis, project administration, data curation, supervision, resources. **Laxsini Murugesu:** investigation, writing–review and editing, formal analysis, methodology, data curation. **Vincent Groot:** conceptualization, methodology, writing–review and editing. **Brigit A. Jong:** conceptualization, funding acquisition, methodology, writing–review and editing.

## Ethics Statement

The Medical Ethics Review Committee of Amsterdam UMC, location VUmc, confirmed that no ethical approval was required for this study (2022.0097).

## Consent

Participants provided consent to participate in the observational study by completing formal written consent. For participation in the interviews, patients gave initial consent verbally and formal written consent before the interview. Participants consented to publication and presentation of the results of the research project.

## Conflicts of Interest

The authors declare no conflicts of interest.

## Supporting information

Supporting information.

Supporting information.

## Data Availability

Data are unavailable as consent was only provided by participants to share information with the research team. Further, complete transcripts and sections of transcripts for individual participants may be identifiable.
