# Supplementary material for: ‘So Let's Go On Like This?’—Shared Decision‐Making and the Use of Outcome Information in Routine Care Management for People With Multiple Sclerosis
Source: Health Expect. 2024 Oct 24;27(5):e70009. doi: 10.1111/hex.70009 (PMC11500206; doi:10.1111/hex.70009)
Supplement: Supplementary file 1 — Supporting information. [file HEX-27-e70009-s002.docx]

**Supplemental file 1: Interview protocol**

**Patient version**

**Introduction (after signing informed consent)**

- Good morning/afternoon. My name is <NAME RESEARCHER> , researcher at the Amsterdam UMC.
- Thank you for being here and for your willingness to participate in this study.
- As explained in the patient information letter, our study aims to assess how different types of information can be used effectively in healthcare to support the process of shared decision-making by the doctor and patient.
- You recently had an appointment with your doctor, <NAME, DATE>, in which she/he discussed the possible treatment of MS with you. We would like to interview you about your experiences with that conversation.
- It is okay if you do not remember exactly what was said in the consultations; I will provide a summary of it.
- It is an open conversation so you can say anything that comes to mind. There are no right or wrong answers.
- This study is intended to learn from it, so we would like to collect all experiences of patients, positive or negative.
- Everything you say during this interview will be treated confidentially.
- I would like to record the interview with an audio recorder. All interviews are processed anonymously into a report, without your personal data being used. The audio file will be deleted after processing the interview.
- The interview lasts between 30 and 60 minutes. If at some point, you become tired, please let us know. Then we can take a break in between or just quit the interview.
- Do you have any questions before we start?

**Standard probing questions during the interview**

- Why do you think that?
- Can you explain that a little more?
- What do you think of that?

**General experiences with the recorded consultation**

You recently had a conversation with your doctor.

- How were you doing at that moment, and how are you doing right now?
- I will first provide a short summary of your conversation with the doctor: <PROVIDE SUMMARY>
- Is my summary correct in your opinion?
- Can you tell us a little bit more about how you experienced the conversation with your doctor?
- In your experience, what went well, what went less well?
- Did you experience any difficulties in understanding information provided by your doctor?

**Use of outcome information**

During the conversation, your doctor discussed information with you.

- (Recall) Is there any information that you specifically remember?
- If yes: What is the information that comes to mind right now?
- If no, <GIVE EXAMPLE FROM CONSULTATION> can you remember how <EXAMPLE INFORMATION> was discussed?
- (Recall) I would like to ask some questions about the information discussed in the consultation. < CHOOSE EXAMPLE, E.G. MRI RESULT, BLOOD TEST RESULT, TREATMENT PLAN, COMPLETED PROM QUESTIONNAIRE>
- What do you remember most about that information?
- Can you explain in your own words what the core message of that information was?
- (Information preferences/needs): Do you, as a patient, need this kind of information? Why/why not?
- Do you plan to do anything now based on that information?
- Do you have any suggestions to improve the information?
- (Communication): What did you think of the way the doctor explained the information?
- Were there any parts you could understand well/less well?

**Experiences /needs Shared Decision Making**

Patients can increasingly participate in decisions made, together with their doctor. For example, you can think about decisions related to the treatment plan, whether something needs to be changed, or a completely new treatment that might be started.

- How do you feel about this idea of Shared Decision Making with your doctor?
- Thinking about the recorded consultation, was there a decision to be made that you remember? <give some time to think about it and, if necessary, ask whether there was something with multiple options including doing nothing, because sometimes patients do not experience something as a choice>
- <If there was choice> How did you experience this process of decision-making?

**Experiences different steps of Shared Decision Making in the recorded consultation**

Shared Decision Making usually takes place in several steps. I will go through each of these steps with you and then I would like to know how you thought this step went during the recorded consultation.

- Step 0 (monitoring and management <SHORT EXPLANATION>): How did you experience this?
- Step 1 (choice <SHORT EXPLANATION>): How did you experience this?
- Step 2 (options <SHORT EXPLANATION>): How did you experience this?
- Step 3 (preferences <SHORT EXPLANATION>): How did you experience this?
- Step 4 (decision <SHORT EXPLANATION>): How did you experience this?

**Evaluation of outcome-information**

- Thinking back of the information the doctor discussed with you, how much did it help you in Shared Decision Making?
- What information do you consider most useful to facilitate Shared Decision Making about options?

**Ending the interview**

- Are there any additional topics that you would like to discuss in relation to this study?
- Do you have any questions or comments regarding the study and/or interview?
- Thank you for your participation!

**Healthcare professional version**

**Introduction (after signing informed consent)**

- Good morning/afternoon. My name is <NAME RESEARCHER> , researcher at the Amsterdam UMC.
- Thank you for being here and for your willingness to participate in this study.
- Our study aims to assess how different types of information can be used effectively in healthcare to support the process of shared decision-making by the doctor and patient. This can be individual, N=1 outcomes, both clinical outcomes and PROMs or aggregated outcome information, e.g. from clinical guidelines.
- As part of our study you recorded one or more consultations with patients at an earlier stage. Today's interview is partly about these consultations and partly about your general experiences.
- It is okay if you do not remember exactly what was said in the consultations; I will provide a summary of it. It is an open conversation so you can say anything that comes to mind. There are no right or wrong answers. This study is intended to learn from it, so we would like to collect all experiences of healthcare professionals, positive or negative.
- Everything you say will be treated confidentially.
- I would like to record the interview with an audio recorder. All interviews are processed anonymously into a report, without your personal data being used. The audio file will be deleted after processing the interview.
- The interview lasts between 30 and 45 minutes.
- Do you have any questions before we start?

**Standard probing questions during the interview**

- Why do you think that?
- Can you explain that a little more?
- What do you think of that?

**General attitudes and experiences with Shared Decision Making**

When discussing a treatment plan with MS patients, there are various situations where ‘there is a choice' and where the patient's voice is important. You may think about decisions related to the treatment plan, whether something needs to be changed, or a completely new treatment that might be started. But also a choice between different types of medication.

- How do you feel about the idea of Shared Decision Making?
- What decisions/situations do you immediately think of when I talk about Shared Decision Making?
- Do you also apply Shared Decision Making in those situations?
- According to you, are there other situations in which Shared Decision Making is suitable?
- A common theme in MS is uncertainty about disease progression , complaints and medication.
- How do you think of this in relation to Shared Decision Making?
- How do you discuss this uncertainty when decisions about the treatment plan are to be made?

**Use of (numerical) evidence /outcome-information**

Traditionally, clinical outcome information such as from RCTs (usually translated into guidelines) has been used as (numerical) evidence to discuss with the patient. More recently, other types of outcome information have also been added, such as patient-reported outcomes (PROMs). Particularly new is so-called N=1 information based on PROMs.

- (How) do you discuss these more classic and newer forms of information with your patients?
- In your opinion, how does this relate to Shared Decision Making?
- Specifically about N=1 PROMS: Do you use N=1 PROMs yourself?
- Why/why not?
- What may be advantages and/or disadvantages?

**Experiences/needs in using outcome-information in different SDM steps**

Shared Decision Making usually takes place in several steps. I would like to show you an overview of the steps. Maybe this is a repetition of what you already know. <SHOW OVERVIEW>

- In which steps in Shared Decision Making do you potentially use outcome-information? Or in which steps would you like to use it?
- What do you think goes well in practice and what may go less well?
- Which problems do you encounter most frequently? How do you deal with these problems?
- What would help you to reduce these problems or otherwise make Shared Decision Making easier?
- What information or other resources would you possibly need in this context?
- How do you experience Shared Decision Making with patients with limited health literacy? <EXPLAIN HEALTH LITERACY IF NEEDED>.

**Evaluation of the recorded consultations**

- A total of X of your consultations with patients were recorded as part of our study. Before I provide a summary of the conversations, I am curious to know to what extent you think the different steps of Shared Decision Making were applied in your consultations?
- <PROVIDE SUMMARY>
- Is my summary correct in your opinion? ?
- Is this how it often goes?
- (example question1): Before discussing the results, you often first ask whether the patient has viewed the results themselves. Why start with that?
- (example question 2): In some cases, the patient initiates choice awareness about, for example, starting/reducing medication or additional diagnostics, such as an MRI scan. Is this typical of conversations with MS patients? What does this mean for Shared Decision Making?
- (example question 3): You asked the patient whether he /she had considered X/Y/Z. The patient immediately has an answer, namely that she does not need this care now. How do you view this? What do you think this means for Shared Decision Making?
- (example question 4): In some cases, the patient's disease course appears stable, but the patient himself/herself indicates that he/she is experiencing complaints. How do you deal with that? What do you think this means for Shared Decision Making?

**Ending the interview**

- Are there any additional topics that you would like to discuss in relation to this study?
- Do you have any questions or comments regarding the study and/or interview?
- Thank you for your participation!
