# Supplementary material for: ‘So Let's Go On Like This?’—Shared Decision‐Making and the Use of Outcome Information in Routine Care Management for People With Multiple Sclerosis
Source: Health Expect. 2024 Oct 24;27(5):e70009. doi: 10.1111/hex.70009 (PMC11500206; doi:10.1111/hex.70009)
Supplement: Supplementary file 2 — Supporting information. [file HEX-27-e70009-s001.docx]

**Patient questionnaire**

1. What is the highest education you have completed?

1. Did not attend or did not complete education
2. Primary school (primary education)
3. Lower vocational education (LBO, LTS, LHNO, domestic/craft school, LEAO, lower agricultural and horticultural education, etc.)
4. Preparatory or short secondary vocational education (VMBO, KMBO)
5. Secondary general education (LAVO, ULO, MULO, MAVO, 3-year HBS, etc.)
6. Secondary vocational education (MBO, MTS, MEAO, Practical Diploma in Accounting, Nursery School, etc.)
7. Secondary general education (5-year HBS, MMS, HAVO, lyceum, atheneum, gymnasium, VWO, etc.)
8. Higher vocational education (HBO, HTS, HEAO, Social Academy, HHNO, teacher education, etc.), candidate examination
9. Scientific education (university)
10. Other, namely………

2. Which form of multiple sclerosis (MS) have you been diagnosed with?

1. clinically isolated syndrome (CIS)
2. relapsing remitting MS (RRMS)
3. secondary progressive MS (SPMS)
4. primary progressive MS (PPMS)
5. I don't know

3. In what year were you diagnosed with multiple sclerosis (MS)?

1. In: ………
2. I do not know (anymore)

(NVS-D): 3. This information is on the back of a container of a pint of ice cream.


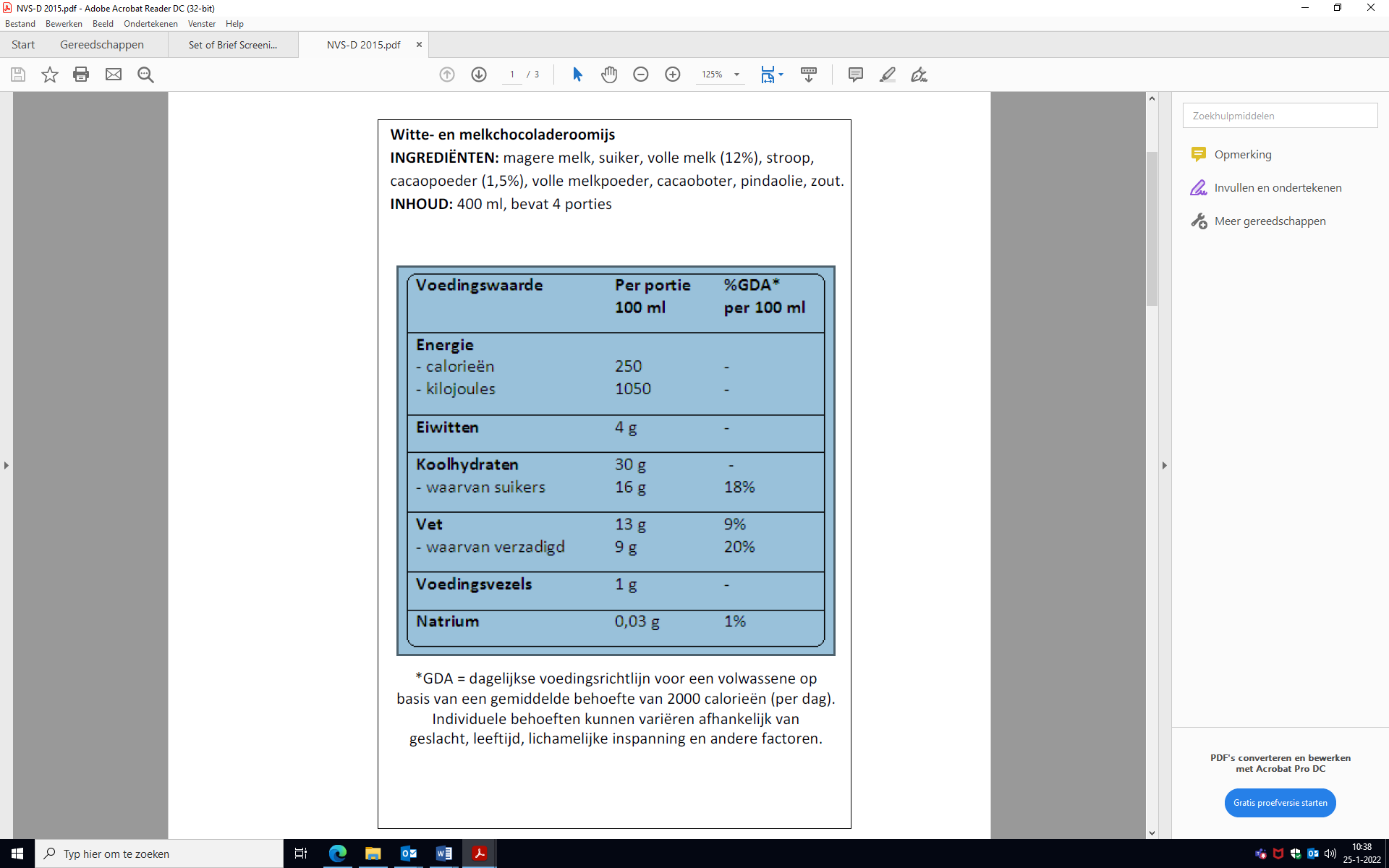


1. If you eat the entire container, how many calories will you eat? …
2. If you are allowed to eat 60 grams of carbohydrates as a snack, how much ice cream could you have? …..
3. Your doctor advises you to reduce the amount of saturated fat in your diet. You usually have 42 g of saturated fat each day, which includes one serving of ice cream. If you stop eating ice cream, how many grams of saturated fat would you be consuming each day? …….
4. If you usually eat 2,500 calories in a day, what percentage of your daily value of calories will you be eating if you eat one serving? ….%

Pretend that you are allergic to the following substances: penicillin, peanuts, latex gloves, and bee stings.

1. Is it safe for you to eat this ice cream? Yes/No

Please only answer the following question if your answer to question e was ‘No’.

1. Why not?

(SBSQ) 4. Please circle the answer that best represents your response.

a. How often do you have someone help you read hospital materials?

1. Always

2. Often

3. Sometimes

4. Occasionally

5. Never

b. How confident are you filling out medical forms by yourself?

1. Not at all

2. A little bit

3. Somewhat

4. Quite a bit

5. Extremely

How often do you have problems learning about your medical condition because of difficulty understanding written information?

1. Always

2. Often

3. Sometimes

4. Occasionally

5. Never
